# Spirituality and Well-Being: Theory, Science, and the Nature Connection

**DOI:** 10.3390/rel12110914

**Published:** 2021-10-21

**Authors:** Carol D. Ryff

**Affiliations:** Institute on Aging, Department of Psychology, University of Wisconsin-Madison, Madison, WI 53706, USA;

**Keywords:** spirituality, eudaimonic well-being, nature, animism, indigenous peoples, Two-Eyed Seeing

## Abstract

The links between spirituality and eudaimonic well-being are examined, beginning with a look at theoretical issues as to whether spirituality is best construed as part of well-being, or as a possible influence on well-being. A brief review of scientific findings from the MIDUS study linking religion and spirituality to well-being and other outcomes is then provided to show recent empirical work on these topics. Suggestions for future work are also provided. The third section is forward-thinking and addresses the power of nature to nurture spirituality and well-being, beginning with a look at how current research has linked nature to human flourishing. Issues of spirituality are rarely mentioned in this literature, despite evidence that nature has long been a source of inspiration in poetry, literature, art, and music. These works reveal that the natural world speaks to the human soul. To explore such ideas, parts of Jungian psychology are revisited: the soul’s longing for poetry, myth, and metaphor; the importance of animism, which sees nature as a field inhabited by spirit; and the devaluing of ancient cultures. The final section considers the wisdom of the indigenous peoples who saw spirit in everything. Their inputs, exemplified with “Two-Eyed Seeing”, offer new visions for thinking about the interplay of spirituality, well-being, and the natural world.

## 1. Introduction

Understanding connections between spirituality and psychological well-being requires theoretical acumen as well as thoughtfully performed empirical science. The need to reach into new territories in examining these linkages is also paramount. With these objectives in mind, the first section below is theoretical in nature and involves a return to the conceptual underpinnings of a model of eudaimonic well-being I put forth years ago (Ryff 1989) that has had widespread impact (Ryff 2014, 2018a). The measures have been translated into 40 languages and more than 1200 publications have been generated. Given the scope of this work, it is useful to consider whether ideas of spirituality were present in the distant formulations drawn on to build the integrative model of well-being. Did any of those past theories emphasize spirituality in distilling core meanings of optimal human functioning? This inquiry illuminates an important theoretical issue: namely, the conceptual distinctiveness versus overlap in conceptions of spirituality and psychological well-being. A fundamental issue is whether spirituality is best formulated as something that constitutes part of what defines well-being, or is better construed as a separate realm that possibly influences aspects of well-being, such as living a life of purpose and experiencing personal growth over time.

Building on these theoretical matters, the second section shifts to consideration of scientific research that has linked religiosity and spirituality to diverse outcomes, including well-being. Here, the contribution is to briefly review wide-ranging empirical findings from a large national longitudinal study, known as MIDUS (Midlife in the U.S.), which I have led for the past two decades. The rich, multidisciplinary data from MIDUS are publicly available and have attracted more than 24,000 users who have contributed to the scientific productivity from the study (www.midus.wisc.edu). In addition to questions about religion and spirituality, MIDUS has comprehensive assessments of psychological well-being (eudaimonic, hedonic), personality, health behaviors, stress exposures, and comprehensive measures of health, including biomarkers. This rich array has afforded numerous scientific advances, offering insights into why some individuals are religious or spiritual and others not, delineating correlates of religion and spirituality, examining how these domains matter for doing good works, and how they are linked with health, broadly defined. Such findings underscore how prospective population research is advancing knowledge of the antecedents and consequents of religion and spirituality. Topics missing in this literature are also considered, which is important given that most people in the general public view themselves as religious or spiritual, or both. These realms thus constitute key features of the human experience.

The third section aspires to be forward-thinking by reaching for new territories in connecting spirituality and human well-being. The specific future direction considered is how encounters with nature may activate and deepen spirituality in ways that enhance eudaimonic becoming. This section examines the growing scientific literature now investigating how nature matters for human flourishing, but notes that spirituality is rarely mentioned in such endeavors. Nature as a longstanding source of inspiration in many art forms, including poetry, literature, music, and painting, is then considered. These works speak to the power of nature to stir up the human soul. Matters of religion and spirituality are thus revisited via how we connect with the natural world. These ideas were evident in the later writings of Jung and are fundamental in world views and practices of indigenous peoples. Our era has undervalued insights from these ancient cultures that offer visions about the interplay of spirituality, well-being and nature. The overarching message is that close connection to and participation in the natural world, including through the arts, may vitally nurture sacred, soulful experiences that help us become our best selves and also activate the caring and concern that is needed to protect the planet on which we live.

## 2. Theoretical Issues: Are Spirituality and Well-Being Distinctive or Overlapping Domains?

The word *soul* seems fundamental to efforts to invoke spirituality. Indeed, the soul is often defined as the spiritual part of being human, while spirituality, in turn, is often defined in terms of the soul, as distinguished from material and physical qualities of being human. I published an article titled “Well-Being with Soul: Science in Pursuit of Human Potential” (Ryff 2018a) and here reflect about the meaning of the word soul in the title. First and foremost, my intent was to draw attention to Aristotle’s assertion in the *Nichomachian Ethics* (Aristotle 1925, 349 B.C.) that the highest of all human goods was “activity of the soul in accord with virtue” (p. 11). This statement captures the essential meaning of eudaimonia, which Aristotle formulated as growth toward realization of one’s true and best nature. The key task in life is thus to know and live in truth with one’s daimon, a kind of spirit given to all at birth. As such, eudaimonia embodies the Greek imperatives of self-truth (know thyself) and striving toward excellence of one’s unique potentialities (become what you are) (see Ryff and Singer 2008). Importantly, nothing in this conception of eudaimonia invoked religious experience or contact with the divine. Rather, the overarching emphasis was on ethical doing.

Centuries later, numerous formulations from clinical, developmental, existential, and humanistic psychology sought to articulate key meanings of positive human functioning (Ryff 1982, 1985). These were woven together in an integrative model of well-being (Ryff 1989). Here, I ask what, if anything, these perspectives had to say about religion or spirituality. Maslow’s (1955, 1968) view of self-actualization emerged from distinctions between deficiency-motivated and growth-motivated needs. He believed the lower-level needs (e.g., physiological requirements for living; safety/security from harm) had to be satisfied before one could move on to self-actualization where the self could be enlarged and enriched. Choosing from among his friends, associates, and historical figures, Maslow examined their qualities and from these, generated characteristics of self-actualized persons, which are many (see Ryff 1985). Most had no linkage to religious or spiritual matters, although two pointed in that direction. Self-actualizers were described as having a continued freshness of appreciation that involved experiencing life with pleasure, awe, and wonder, which some might see as spiritual. In addition, some self-actualizers had mystical or peak experiences characterized by intense ecstasy, bliss, and awe wherein the self was transcended, and one was gripped by feelings of power, confidence, and decisiveness. Maslow saw these happenings as akin to religious experience.

Rogers’ (1961) conception of the fully functioning person emerged from his work with people in therapy. Like Maslow, he believed the person would grow naturally, if freed from psychological defenses and external impediments. His characteristics of the fully functioning person gave no emphasis to the spiritual realm, although openness to experience (i.e., living fully in each moment and not seeing in preconceived categories) seems similar to Maslow’s emphasis on freshness of appreciation. Allport’s (1961) conception of maturity included numerous characteristics of the mature person, such as emotional security, warm relating to others, and realistic perception. These had no connection with spirituality, although the final feature of mature persons was that they had a unifying philosophy of life. This quality meant having a clear comprehension of life’s purpose, a sense of directedness, and intentionality. Notably, Allport observed that this unifying philosophy of life for some was related to religious sentiment.

Erikson’s (1959) model of ego development included stages of continuing growth (e.g., formulating an identity in late adolescence, experiencing intimate connection in early adulthood, caring about guiding the next generation in middle adulthood), none of which invoked spirituality. However, his final stage of ego integrity encompassed multiple things: emotional integration, acceptance of one’s past life, feeling a comradeship with distant times, and having a love of humankind more than of the self. He also conveyed that integrity involved achieving a spiritual sense, which eliminated the fear of death. Another developmental approach focused on life tendencies that work toward fulfillment (Bühler 1935; Bühler and Massarik 1968). These emphasized being active and productive in the pursuit of one’s self-development, but no emphasis was given to spirituality as part of such fulfillment.

The major contribution from existential psychology was Frankl’s emphasis on purpose in life (Frankl 1959; Frankl and Lasch [1959] 1992). The critical task is to find meaning in one’s life, including in contexts of suffering, or in the face of a world that seems meaningless. Importantly, Frankl’s formulated logotherapy and religion as separate realms, though he acknowledged that spiritual beliefs may make it easier for some to find meaning (Okan and Ekşi 2017). He acknowledged diverse religious orientations, but also believed one could find meaning without faith. Frankl himself was religious, as was his wife, a Catholic. They respected the practices of their respective faiths.

The Jungian perspective on individuation (Jung 1965; Von Franz 1964) emerged from intense self-examination including scrutiny of his own personal crises. Strong emphasis was placed on the unconscious as a way of achieving self-development. Jung stated he could not “employ the language of science to trace this process of growth in myself, for I cannot experience myself as a scientific problem” (Jung 1965, p. 3). He felt that reason and rationalism set the boundaries too narrowly for us. Of relevance for adult development were distillations of different motivations and meanings from early and middle adulthood (Jung 1933). The importance of accepting and expressing both the masculine and feminine features of oneself was also emphasized. Jung believed self-realization required a deliverance from convention—those clinging to the collective fears, beliefs, laws, and systems of the masses would not become fully individuated. Importantly, the journey required seeing one’s dark side—the shadow within oneself. Overall, Jungian individuation involved working toward a harmonious integration of all aspects of the self, including the unknown and the mysterious.

Jung wrote extensively about religious and spiritual matters (Tacey 2013), observing that secular society had degraded the *pneuma*—what the Stoic philosophers saw as the vital spirit, soul, and creative force of the person. He lamented the rise of religious fundamentalism, a topic to which I will return to in the empirical section. Drawing on German Romanticism, which included much beautiful poetry about nature, he also saw the connection between the ecology of the soul and the natural world (Tacey 2013). These ideas are elaborated in the future directions section.

Not included in the above theories that comprised the integrative model of well-being, but pertinent to this inquiry is William James ‘ ([1902] 1958) *The Varieties of Religious Experience*. His remarkable chapters on “healthy-mindedness” and the “sick soul” captured with eloquence the upsides and downsides of psychological experience—as tied to religion. Those who are healthy-minded had souls of a sky-blue tint that allowed them to benefit from the conquering powers of courage, hope, and trust. They also had contempt for doubt, fear, and worry. Alternatively, there were the morbid-minded who could not throw off the consciousness of evil. These children of wrath knew that good things would perish, that fame, riches, youth, and health would vanish and ultimately, “the skull will grin at the banquet” (Ryff 2018b, p. 397). These descriptions set the stage for subsequent research on many topics, including hope, optimism, and self-efficacy for those who live on the sunny side of the street, along with work on the depressed and anxious who are closer to the pain threshold, with lives played out in darkness and apprehension. His insights about these two forms of being were surely tied to his own experiences, which included leading a rich, creative life while struggling repeatedly with depression (Simon 1998). James saw normal personal development as involving the unification of these two selves, but noted the journey was not always successful.

Several points follow from this return to the theoretical foundations of eudaimonic well-being (Ryff 1989), with a detour through William James along the way. The first is to observe that most of the proffered meanings of positive psychological functioning did not involve matters of religion or spirituality. As such, the six key dimensions of well-being—i.e., autonomy, environmental mastery, personal growth, positive relations with others, purpose in life, self-acceptance—identified as points of convergence in these perspectives (Ryff 1989) *did not encompass spiritual matters.* The structured self-assessments scales created to operationalize these six dimensions also *did not focus on spirituality*. The lack of emphasis on spirituality could be seen as theoretical limitation, but it can also be viewed as a scientific strength. Scientific efforts to link spirituality and well-being are less fraught with tautologies if each domain is defined and measured as separate and distinct from the other. The next section examines empirical findings linking religion and spirituality with diverse aspects of well-being. That theoretical and measurement boundaries between these realms are not blurred is a scientific plus.

Some additional points from this past work (Ryff 1985) are worth iterating. One is the misguided claim coming from several theorists that personal growth is innate and will follow naturally if impediments are removed. I thought then and continue to believe this assertion is problematic, citing Smith’s (1974) observation that “vice and evil are as much in the range of human potentiality as virtue, specialization as much as well-rounded development. Our biology cannot be made to carry our ethics, as Maslow would have it” (p. 172). Seeing personal growth as biologically based also deflects attention away from the actual experiences of people’s lives, the pivotal events, sometimes traumatic, that may catalyze discovery of unknown resources and self-knowledge. Another point worth repeating pertains to historical and cultural variation in conceptions of ideal human functioning where my primary source was Coan’s (1977) tour de force look across centuries of human history. His central message was that meanings of optimal, ultimate human experience are not universal: the ancient Greeks placed heavy emphasis on cultivation of the human mind guided by reason, while writers from the Middle Ages emphasized saintliness and close contact with the divine, along with concern for the welfare of others and unselfish love. The Renaissance brought a new spirit of creativity and individual expression to ideals of human functioning with less appeal to authority in matters of thought, morality, and taste.

Coan further contrasted Western and Eastern views of human fulfillment, reminding the reader that rationality has been more dominant in the West while intuition is more conspicuous in the East. The West also sees the person as having a separate reality, whereas Eastern perspectives, such as among Hindus and Buddhists, see less separation of the individual from the whole of nature. Indeed, the goal of Hinduism involves a loss of experienced separateness and achieving a sense of oneness with the universal soul. There is also greater emphasis on suffering, routed in desire, in Buddhism, while Taoism focuses on the relationship of the individual to the whole of nature via attending to the mystical, the intuitive, and letting things take their course. Modern Indian thought exemplified by Sri Aurobindo emphasized evolution of the soul and shifting away from reason or intellect toward a more spiritual being. Taken as a whole, this wide sweep of human ideals across time and context contrasts with universals now emphasized in positive psychology (Peterson and Seligman 2004).

A final theoretical observation pertains to issues of fragmentation versus wholeness in conceptions of psychological well-being and spirituality. On the one hand, eudaimonia (theoretically and empirically) is notable for blending experiences of thinking, feeling, and striving, thus transcending divisions in psychology that partition the person into separate realms of cognition, emotion, and motivation. At the same time, the differentiation of distinct dimensions of well-being creates another kind of fragmentation, given that most scientific work examines these characteristics one at a time rather than considering whole profiles of wellness (Pancheva et al. 2020). For better or worse, scientific efforts to understand often proceed by differentiating the phenomena into component parts, which are then linked to other component parts (e.g., diverse aspects of health).

Such endeavors are antithetical to the ideas of wholeness now receiving heightened attention (Niemiec et al. 2020; Russo-Netzer 2018), as they should. Features of wholeness have been delineated to include embracing life with breadth and depth, having a life affirming view of oneself and the world, and being able to organize one’s life journey into a cohesive whole. Wholeness also encompasses complexity, paradox and dichotomies as well as ideas of brokenness. The juxtaposition of wholeness and brokenness (Pargament et al. 2016) highlights several additional distinctions: purposive vs. aimless; broad and deep vs. narrow and shallow; flexible and enduring vs. rigid and unstable, balanced, cohesive, and discerning vs. unbalanced, incohesive, and non-reflective). These ideas have discernable value in clinical work, counseling, and education, although they are less evident in scientific work that continues to reflect a largely fragmented approach with the whole person almost never in view. A worthy objective in research on spirituality and well-being going forward is to better capture human wholeness.

## 3. Religion, Spirituality, Well-Being and Health: A Review of Scientific Advances from MIDUS

A large prior literature has examined links between religion and health (e.g., Idler 2014; Koenig et al. 2012; Li et al. 2016). Given this broad field, it is relevant to ask why to focus on findings from a single study? Two justifications are offered for emphasis on the Midlife in the U.S. (MIDUS) national longitudinal study. First, a central objective of MIDUS is to examine human health and well-being as an *integrated biopsychosocial process* (Ryff and Krueger 2018). That is, the overarching commitment is to work across disciplinary domains. This means that MIDUS not only includes detailed measures of religion and spirituality pertinent to this Special Issue, but also has unusual depth in assessments of psychological and social factors, health behaviors, life stressors and health, including a rich array of biomarkers. These measurement strengths, along with the longitudinal design and large, national samples, afford unique opportunities to sharpen the understanding of relations between religion and spirituality with well-being and health.

Second, MIDUS data are publicly available and have been widely used by researchers all over the world to generate more than 1500 publications (see www.midus.wisc.edu). Importantly, no permissions are required to use the data, which are notable for being well-documented and user-friendly. Thus, numerous opportunities exist for others to engage with the expansive MIDUS data. As will be noted following a look at illustrative findings, many important questions have yet to be examined.

As a prelude to findings, it is noteworthy that most MIDUS participants consider themselves to be spiritual. When asked in 2002 (the second wave for the core sample) “How spiritual are you?” most participants answered a lot (30%) or somewhat (46%). Similarly, when asked “How important is spirituality in your life?” most answered a lot (49%) or somewhat (34%). The same questions were asked in 2012 for the Refresher sample, involving recruitment of a new national sample of same-aged adults (25 to 74) as the baseline sample recruited in 1995. The majority of Refresher participants also considered themselves to be spiritual and considered spirituality as important to them. In contrast, less than a third (30%) of both samples reported that they attended weekly religious or spiritual services. Thus, although spirituality and religious practice were not strongly linked, spirituality is prominent in the self-perceptions of most MIDUS participants who represent the general population in the U.S.

To date, over 60 publications have been generated on religion and spirituality using the MIDUS data (www.midus.wisc.edu/publications/), some noted here. One interesting question is what accounts for why some people are religious and others not. Bierman (2005) examined links between physical and emotional abuse in childhood, with adult religiosity. The key finding was that abuse committed by a father predicted less religiosity, although abuse outside the immediate family was linked with increased spirituality. A possible explanation was that victims of abusive fathers may distance themselves from images of God, the father. Alternatively, Jung (2018) found that the effects of childhood adversity on adult mental health were reduced among those who were involved in religious practices—specifically, religious salience and spirituality buffered the noxious effects of child abuse on changes in positive affect over time. Other studies examined the psychological correlates of religiosity. Greenfield et al. (2009) found that higher levels of spirituality were associated with higher levels of well-being (positive affect, purpose in life, positive relations with others, personal growth, self-acceptance, environmental mastery, autonomy), with some associations stronger among women than men. Greater religious participation was also linked with higher purpose in life and personal growth, but also with lower autonomy. Having a religious social identity was found to mediate the link between more frequent attendance at religious services and higher levels of hedonic well-being (more positive affect, less negative affect, more life satisfaction) (Greenfield and Marks 2007). A further mediational study showed that psychological resources (emotional and psychological well-being) mediated the effect of early exposure to religion on self-rated health and physical symptomatology (Son and Wilson 2011). Another inquiry found that religiosity and personality characteristics (agreeableness, conscientiousness) were uniquely associated with cognitive coping (optimism, positive reappraisal) (Schuurmans-Stekhoven 2018). Whether religious beliefs compensate for purpose in life among the socially disconnected was examined (Chan et al. 2019) with findings showing that religious beliefs had minimal influence on purpose in life among socially connected individuals, but for those who were socially disconnected, being highly religious predicted higher levels of purpose in life.

Does religiosity contribute to good works, such as volunteering, helping others, and prosocial behavior? Taniguchi and Thomas (2011) found that religious inclusiveness (being open to other faiths) promoted both religious and secular volunteering, whereas religious exclusiveness promoted volunteering only in religious areas. Einolf (2013) found that daily spiritual experiences were significant predictors of volunteering, charitable giving, and helping others. Gender differences emerged as correlates of volunteering and charitable giving, with women showing broader social networks through religious participation (Einolf 2011).

Multiple inquiries have linked religion and spirituality to health. Issues of directional influence have been examined, with McFarland et al. (2013) finding that a cancer diagnosis was associated with increased religiosity, as well as strong evidence that people diagnosed with cancer at earlier ages experienced the largest increases in religiosity over time. Others found that high spiritual experiences enhanced life satisfaction over time in cancer survivors having low satisfaction at baseline (Rudaz et al. 2019). Additional work showed that spiritual coping influenced the personal growth of cancer patients, with effects moderated by spiritual mindfulness (Rudaz et al. 2018). After controlling for multiple factors, religious and spiritual identities were found to predict greater use of complementary and alternative medicine (Ellison et al. 2012).

With regard to health behaviors, high levels of religious and spiritual involvement were found to predict lower odds of smoking over time (Bailey et al. 2015). Addiction recovery programs such as Alcoholics Anonymous are known to promote spiritual but not religious beliefs. Reflecting this emphasis, McClure and Wilkinson (2020) showed that frequency of attendance in such groups was positively and significantly associated with being spiritual but not religious. Links between religion and body weight have been of interest. Kim et al. (2003) found that religious denomination was significantly related to body weight after accounting for sociodemographic controls: specifically, conservative Protestant men had a higher body mass index (BMI) than those reporting no religious affiliation. A related study (Kim 2007) showed that women and men with greater religiosity were more likely to underestimate their body weight. Some studies have linked religiosity to biomarkers: Tobin and Slatcher (2016) found that higher levels of religious participation predicted steeper (healthier) profiles of diurnal cortisol after controlling for confounds. Finally, links between religion and mortality (length of life) have been examined. Upenieks et al. (2021) found that children brought up in highly religious households had higher risk of mortality than those socialized in moderately religious households.

Several summary observations follow from these findings, most of which control for numerous covariates and relevant confounds. Although religion and spirituality have typically been studied as positive influences on diverse aspects of well-being and health, some findings have shown negative effects—conservative Protestant men showed higher obesity profiles than non-religious men, while children brought up in highly religious households had higher mortality risk than those raised in moderately religious households. Opportunities to investigate the possible costs of high religiosity and spirituality remain. Why is this worth pursuing? Primarily because religion is known to have both helpful and harmful effects (Pargament 2002). These bitter and sweet fruits were recognized long ago by William James ([1902] 1958) and were also addressed by Jung in writing about the spiritual problems of modern man (Jung 1933; Tacey 2013). They also emerged in Allport’s (1950) classic work, finding that average churchgoers were more prejudiced than non-churchgoers. This observation led to the distinction between intrinsic versus extrinsic religious motivations (Allport and Ross 1967; Donahue 1985)—i.e., the difference between embracing and internalizing the creed and values of one’s religion, as opposed to using one’s religion for personal ends, such as seeking status, security, and sociability. Prejudice was found to be more common among the latter. Recent empirical work has linked religious fundamentalism to multiple negatives: authoritarianism, narrow mindedness, discrimination, and bigotry (Altemeyer and Hunsberger 1992; Hood et al. 1996; Kirkpatrick 1993). Our contemporary world makes it undeniable that religious convictions sometimes translate to hate, intolerance, and even the killing of others.

MIDUS cannot address these extremes, nor does it offer insights into religious motivations. That said, the study has high-quality measures of perceived discrimination, which illuminate who among the sample see themselves as recipients of prejudice and unjust treatment. A first, highly cited study (Kessler et al. 1999), reported on the prevalence, distribution, and mental health correlates of perceived discrimination. More than 80 additional publications subsequently linked assessments of daily and lifetime discrimination to mental and physical health outcomes. Participants report the reasons they perceive for receiving unfair treatment, which include their race or ethnicity, gender, age, socioeconomic status, religion, sexual orientation, and weight. Thus, significant avenues for future inquiry could investigate links between religious beliefs and practices and perceptions of discrimination.

MIDUS also has extensive measures of stress exposures, such as chronic and acute life events, caregiving responsibilities, and daily hassles. Measured at each wave, these afford empirical handles on cumulative adversity across time. Relevant questions are whether aspects of religion and spirituality serve as buffers (moderators) of the effects of wide-ranging life stressors on well-being and health. Religion may be particularly valuable when people face problems that push them to the limits of their own personal and social resources, thereby exposing basic vulnerabilities (Pargament 1997). MIDUS also has depth in assessing social relational ties (within and outside the family), including frequency and quality of contact with numerous others. How are religious and spiritual orientations linked with relational well-being? As noted above, religion may nurture purpose in life among those who are socially isolated. Further inquiries could probe whether and how the quality of parent/child relational ties vary by religious experiences as well as how religion and spirituality matter for marital quality, including persistent marital conflict over time. Many MIDUS findings have examined positive and negative aspects of marital quality as well as on spousal loss. Different marital statuses (married, single, divorced, widowed) have also been linked with psychological well-being. Other extensively studied topics include family caregiving and the interplay (both positive and negative) between work and family life. Whether religious and spiritual orientations matter these topics are promising future topics.

Finally, MIDUS has become a major forum for studying ever-widening inequality in the U.S., which has now been heightened by the hardships of the pandemic (deaths, unemployment, hunger, evictions) (Ryff forthcoming). These multiple forms of suffering and their consequences for health and well-being need scientific attention. Many factors (psychological, social, behavioral, biological) are being investigated as possible protective characteristics (buffers) or vulnerabilities (exacerbating influences), but largely unexamined in health inequalities research are influences of religion and spirituality. Stated otherwise, human suffering is now widespread, making its consequences for how well and how long people live a major scientific imperative of our time. Ties therein to matters of spirituality need scholarly attention.

## 4. The Power of Nature to Nurture Spirituality and Well-Being

This section covers three topics: (1) the current science that is now examining how nature contributes to human flourishing; (2) input from the arts and humanities (poetry, literature, music, art, history, philosophy) that showcase nature as a key source of inspiration in life; (3) the spiritual significance of the natural world, drawing on Jungian writings about the soul and the sacred, as well as world views from indigenous peoples. This mélange constitutes a reaching toward ancient and new forms of spirituality about how nature influences our well-being and how we must care for the planet.

Vibrant research is now underway studying how nature contributes to human flourishing (Capaldi et al. 2015; Mantler and Logan 2015). These ideas have growing salience as greater segments of the world’s population live in nature-impoverished urban milieus. Multiple theories have been offered to understand how we benefit from nature. From evolutionary thinking comes the biophilia hypothesis, which suggests that our human ancestors depended on connecting with nature to survive (Kellert and Wilson 1993), along with stress-reduction theory (Ulrich et al. 1991), which proposes that past exposures to unthreatening natural environments contributed to survival via stress-reducing physiological responses (e.g., pulse rate, cortisol levels, immune functioning). Focused not on the distant past but the present is attention restoration theory (Kaplan and Kaplan 1989), which addresses the executive resources needed for cognitive performance, suggesting that natural environments provide opportunities to escape from life’s demands, thereby improving capacities for concentration and attention. Other perspectives consider the role of the natural environment in addressing existential anxieties, such as meaning in life, isolation, freedom, and death (Yalom 1980). Eco-existential positive psychology (Passmore and Howell 2014) thus describe how restorative experiences with nature might contribute to a sense of identity, multiple forms of happiness, meaning, social connectedness, freedom, and awareness of one’s own mortality.

Empirical evidence has linked encounters with nature to high hedonic well-being (positive emotions, less negative emotion, life satisfaction), both short and long-term, and to aspects of eudaimonic well-being, such as meaning, autonomy, vitality, and feelings of transcendence (Capaldi et al. 2015; Mantler and Logan 2015; Triguero-Mas et al. 2015). Some inquiries have examined intervening mechanisms, such as increased physical activity, increased social contact, stress reduction and restoration of cognitive attention. Other literature from environmental psychology examines how natural and built environments promote human capacities (De Young 2013; Gifford 2014; Proshansky 1987). The focus on green spaces in these works underscores growing concerns about urbanization, loss of biodiversity, and environmental degradation. The increasingly dire consequences of climate change (droughts, wildfires, floods) have also led to research on pro-nature behaviors that support the conservation of nature and biodiversity. Richardson et al. (2020) conducted an innovative population survey in the United Kingdom, showing that pro-nature actions were strongly predicted by knowledge of and concerns about nature.

Interestingly, spirituality is rarely mentioned in the above literatures, even though ideas about nature as sources of inspiration and uplift are omnipresent in the human story, as expressed in poetry, literature, music, art, history, and philosophy. An example is the life of Alexander von Humboldt (1769–1859), written about in *The Invention of Nature* (Wulf 2016). Primarily a scientist, naturalist, and explorer (of South America and Siberia), Humboldt influenced many great thinkers of his day, including Jefferson, Darwin, Wordsworth, Coleridge, Thoreau, and Goethe. He was ahead of his time in thinking about the degradation and exploitation of nature, warning that humankind had the power to destroy the natural environment. Humboldt had a sense of wonder about nature, believing that our responses to it should emanate from our senses and emotion—nature, he thought, spoke to humanity in a voice “familiar to our soul” (p. 61). Scenes from mountain tops activated his imagination and soothed the “‘deep wounds’ that pure ‘reason’ sometimes created” (p. 97). Aligned with Goethe’s inner circle of German Idealism and Romanticism, Humboldt saw no irreconcilable chasm between the internal and the external world. As such, he embraced the rationality and methods of the Enlightenment thinkers, while seeing nature, not as an external mechanical system, but as a living organism that required subjectivity. Both Goethe and Humboldt advocated for the marriage of art and science rather than seeing them as antagonists.

How can nature, as expressed in the arts, nurture the subjective parts of who we are and hope to become? Insights come from Mark Edmondson (2004, 2015), who teaches great literature and poetry to nurture well-being, including the values needed by the human soul, such as courage, contemplation, and compassion. When educating college students about great works, he repeatedly asks: Can you live it? Does the work offer a new or better way of understanding the self and others, or point to alternative paths for living a better life? To illustrates, Edmondson examines Wordsworth’s famous poem, “Lines Composed a Few Miles from Tintern Abbey”, written in 1798. The context is that his life had become flat—“he lived in a din-filled city, among unfeeling people, and sensed that he is becoming one of them … there is a dull ache settling in his spirit” (p. 57). Returning to a scene from his childhood, Wordsworth remembered himself as a young boy, free and reveling in nature. The return to nature, which is the heart of the poem, reminded him of its role in nurturing his own vitality. The poem “enjoins us to feel that it (the answer to one’s despondency) lies somewhere within our reach—we are creatures who have the capacity to make ourselves sick, but also the power to heal ourselves” (p. 49).

Wordsworth’s poetry served a vital function in the life of John Stuart Mill ([1893] 1989), who as a young adult realized that he lacked the happiness central to the utilitarian philosophy in which he was steeped. Reflecting on his past, Mill described an early educational experience that was exceptional, but profoundly deficient. His father began teaching him Greek and Latin at a young age and then expanded the pedagogy to fields of philosophy, science, and mathematics. Sentiment and emotion were nowhere to be seen—they were deeply opposed by his father. To escape the logic machine he had become, Mill began a quest to feel, and it was the poetry of Wordsworth, mostly about nature, that ministered to the longings in his soul. He credited it for helping him recover from the crisis in his mental history.

Nature is powerfully present in most art forms. On the heels of the Romantic era in literature came the French impressionist movement (1860–1910). After centuries of religious art, mostly dark and dreary in content, the impressionists began to paint outside (*en plein air*), leading to subject matter suffused with light—the sun shining down on all manner of nature’s beauty. This new vision brought Monet’s famed *Waterlilies* and *Poppies*, and his *Garden at Giverny*. Cezanne captured the *Forest*, Sisley the *Fog*, and Pissarro the French countryside (*Paysage aux Patis)*. Van Gogh dazzled the world with his *Starry Night* and *Sunflowers,* while Klimt, known for figurative art, created breathtaking scenes from nature (*Beech Forest* and *Fruit Trees)*. From Spain, Sorolla captured seascapes (*Biarritz Beach)* and children frolicking in waves (*Niños en el Mar)*. Taken as a whole, the world came to love this art for its magnificent celebration of nature that brought joy and inspiration to all.

Others drew on nature to inspire musical creativity, such as Debussy’s symphonic sketches (*La Mer*) that captured the changing moods, rhythm, and power of the sea. Nature inspired Beethoven’s *Pastorale* symphony, Chopin’s *Raindrop* prelude, Rimsky-Korsakov’s *Flight of the Bumblebee*, and Smetana’s symphonic poem about a beloved river, *The Moldau*. These works continue to evoke rich emotions in others more than a century later. What about contemporary art forms? The 2019 Pulitzer Prize in Literature was awarded to Richard Powers (2018) for *The Overstory*, a novel about the impact of giant, memorable trees on the lives of several people. The 2013 Pulitzer Prize in Music was awarded to John Luther Adams for his orchestral work *Become Ocean*, which “immerses the listener in a sonic churn, ebb and roar that conjures a world inundated by rising sea levels” (Fonseca-Wollheim 2020). Adams thus combines musical composition with environmental activism, a theme also present in Power’s book. Contemporary poetry about nature, evident in the works of Mary Oliver (2017) and Wendel Berry (2012), surely ministers to the souls of many. These are poets follow the steps of one of America’s greatest poets, Walt Whitman, who in *Leaves of Grass* (Whitman [1855] 2005) wrote:

“I believe a leaf of grass is no less that the journeywork of the stars,

And the pismire [ant] is equally perfect, and a grain of sand,

and the egg of the wren

And the tree-toad is a chef-d’oeuvre for the highest,

And the running blackberry would adorn the parlors of heaven.”

(p. 31)

What is the relevance of these expressions and preceding art forms for the theme of this essay—namely, to explicate how nature might matter in connections between spirituality and well-being? On the one hand, there is the prosaic point that science needs to track how often (frequency) and how deeply (intensity) we interact with nature. This is being undertaken in some of the research described above, but not included in those endeavors are the ways in which nature is taken in via literature, poetry, art, music, and film. Both realms of experience are needed complements to topics routinely assessed in studies of health and well-being, such as what people eat (nutrition surveys), drink (alcohol intake), whether they smoke, how often they exercise, and so on. Participation with nature must be recognized as a domain of vital nourishment taken in by many on their journeys through life. There remains, however, the need to illuminate matters of spirituality at the heart of the nature experience. Those topics require a return to select writings from Jung, along with input from indigenous peoples.

David Tacey’s (2013) *The Darkening Spirit: Jung, Spirituality, and Religion* brings new insights to what we can learn from Jung’s extensive writings. The son of a Christian minister, Jung saw numerous problems with conventional religion, including its fundamentalist tendencies, sometimes leading to violence and fanaticism. He also saw the literal and absolutist claims of Western religion as too narrow and out of touch with what the soul longed for: poetry, myth, metaphor, and imagination. Drawing on these ideas, Tacey sees spirit as the expression of what is best and highest in our humanity, and speculates that secularism has, paradoxically, made us more, rather than less, spiritual. In thinking about these matters, we learn that Jung reflected about his own daimon, writing that “it overpowered me, and if I was at times ruthless it was because I was in the grip of the daimon …. I had to catch up to my vision … while my contemporaries saw only a fool rushing ahead” (p. 41). Importantly, Jung used the word ‘soul’ not in the theological sense, but with meanings coming from Greek philosophy.

Reflecting on modern times, Jung saw the 20th century as the most violent, evil, and appalling period in the history of civilization and believed that evil could only be reduced by digging into the shadow. It was in dealing with the dark side that he saw the need to draw on the arts, literature and philosophy, which he believed were inspired by archetypal impulses. Art thus becomes the critical source and expression of spirit in our contemporary world and requires that we give back to the poet, artist, and philosopher their sacred status. Key to searching for a new balance between the dark and the light is recognizing the importance animism, defined as the “experience of nature as a field inhabited by spirits that animate it” (p. 115). Because we have devalued ancient cultures, we have been left in a wasteland where imagination and myth are seen as escapes from, rather than windows into, reality. What is required thus is that we deconstruct the Western imperialist project with its mischaracterizations of so-called primitive peoples. As stated by Tacey: “We dismiss ancient visions as unscientific, but our rational approach may be missing more than we realize. We like to debunk the notion that earth has spirit, and demand that those who make such assertions provide some proof. But we might as well be fishes of the sea, asking proof of the existence of the ocean” (p. 116). Importantly, the failure to see nature as sacred has led to our degradation and exploitation of it.

In reflecting about how the human soul could have become so divorced from the spirits of our natural surroundings, Tacey draws on Hillman’s (1995) foreword to *Ecopsychology*:

Even the high intellectualism of the Renaissance, to say nothing of the modes of mind in ancient Egypt and Greece or contemporary Japan, allowed for the animation of things, recognizing a subjectivity in animals, plants, well’s springs, trees, and rocks. Psychology, so dedicated to awakening human consciousness, needs to wake itself up to one of the most ancient human truths: we cannot be studied or cured apart from the planet.

(p. 125)

The crux of the matter in Tacey’s view is that we need a “down to earth and existential expression of the religious impulse and one that does not fly in the face of science” (p. 143). Without spiritual nourishment and cultural life, the soul loses its way, becomes lost in materialism and worldliness, or afflicted by neuroses and illnesses. Even so, the soul makes appearances in other guises—the arts, cinema, music, and popular culture. Overall, Tacey offers a broadened conception of soul as a force that links us with meaning, while striving for community and seeking connection with nature and ultimate reality.

Tacey has also written about mythic bonds with nature found in Aboriginal Australian cultures. In *Edge of the sacred: Jung, psyche, earth* (Tacey 1995), the spirit and soul of the earth are deeply examined. Science and rationality may dismiss such ideas as illusions, but he reminds the reader that failing to embrace the meaning of an animated earth will leave us alienated and alone, while at the same time contribute to disrespect of and damage to the environment. The wisdom of indigenous peoples has also been emphasized by the Canadian Institutes of Health Research (CIHR) via focus on First Nations, Inuit, or Métis communities and their cultures, experiences, and knowledge systems. This inquiry has led to *Etauptmumk*, translated as “Two-Eyed Seeing” (Roher et al. 2021). This recent review distilled seven categories of meaning in Two-Eyed Seeing: (1) guide for life—i.e., a wholistic way of knowing, being, doing, and seeing that is mental, spiritual, physical and emotional; (2) responsibility for the greater good—i.e., calling for use of all capacities, gifts and actions to leave the world a better place; (3) co-learning journey—i.e., relationship building by having different peoples put their own knowledge and action forward for examination; (4) diverse perspectives—i.e., respecting and accepting diverse realities; (5) spirit—i.e., there is spirit in everything and essential for a complete person is interaction of body, mind, soul, and spirit with all aspects of nature; (6) decolonization—i.e., honoring indigenous perspectives in how knowledge is created, gathered, and used; (7) humans as part of ecosystems—i.e., human health requires balance and integrity between people and the global ecosystems that surround them.

These contributions from indigenous peoples are arguably the most powerful examples we have of how to grasp the meanings of spirituality in the natural world around us, of which we are a part, as well as how to live with a commitment of responsibility to protect all of it. The great tragedy is the scope of trauma inflicted on these cultures by so-called enlightened newcomers. These horrific tales continue to be told, illustrated by *Empire of the Summer Moon* (Gwynne 2010), which details the rise and fall of one of the most powerful tribes in American history, the Comanches. They lived in a world alive with spirits, which were everywhere, in the rocks, trees, and animals. They danced to celebrate these spirits and made offerings to them, but in the end they were decimated by destruction of the buffalo herds, much of which the white invaders carried out maliciously, not for the hides, not for the meat, but with the sole intent of destroying the livelihood of the Plains Indians. Another tale of trauma (Vaillant 2005) comes from the Pacific Northwest, the Haida peoples from the Queen Charlotte Islands, whose lives were greatly harmed, first by the colonialist devastation of the sea otters, followed by widespread clear-cutting of the magnificent forests on which they relied for many things, including to build their massive cedar canoes. The central drama in this book, however, revolves around the cutting of a giant golden spruce, considered sacred by the Haida. Together, these publications constitute forms of conscious-raising, not only about the abhorrent treatment of First Nations peoples, but also about their wholistic ways of being that stand in marked contrast to Western rationalism and science.

## 5. Conclusions

The scope of what has been covered in this article is wide—perhaps so expansive as to be at the edge of incoherence. Is going from a look back at the theoretical foundations of eudaimonic well-being through a review of current science linking religion and spirituality to well-being and health and then onto the possible centrality of nature in understanding deeper meanings of what is sacred and what the soul needs, more than can be meaningfully managed? I hope not. Having studied human well-being for decades and invested much into examining how it matters for health, I believe the next great leap is to embrace the spiritual realm. That can be achieved in many ways, as illustrated by some of the work I have summarized. Nonetheless, reaching still farther toward nature seems fundamentally important because it provides windows on spirituality that exist outside conventional religion and current science, despite having been vital in the history of our species. My objective has thus been to articulate, drawing on a wide range of literatures, how we might embrace a conception of spirituality anchored in nature that may be critical for what we, as individuals, as well as what our planet, currently need.

## Data Availability

The MIDUS data are publicly available and information about accessing them is provided on the website: www.midus.wisc.edu.
